# Real‐World Evidence on Mobile App–Supported Diabetes Management in Insulin‐Treated Patients

**DOI:** 10.1155/jdr/6671361

**Published:** 2025-12-29

**Authors:** Lena Roth, Julian Stein, Peter E. H. Schwarz

**Affiliations:** ^1^ Department for Prevention and Care of Diabetes, Faculty of Medicine Carl Gustav Carus, Technische Universität Dresden, Dresden, Germany, tu-dresden.de

**Keywords:** diabetes mellitus, digital health intervention, eHealth, HbA1c, real-world data, real-world evidence, self-management

## Abstract

**Background:**

Digital health interventions, such as mobile diabetes apps, are aimed at supporting glycemic control. Real‐world data (RWD) provide valuable insights into their long‐term effectiveness beyond the controlled conditions of randomized trials.

**Objective:**

This study evaluates the effectiveness of the mobile diabetes app ESYSTA in improving glycemic control using real‐world evidence.

**Methods:**

A retrospective analysis of 475 users was conducted to assess HbA1c changes after 6 months of usage and also long‐term usage (12 and 15 months). A linear mixed model was used to adjust for confounding factors.

**Results:**

After 6 months, ESYSTA users achieved a significant HbA1c reduction of −0.59 (−0.70; −0.48) percentage points compared to baseline, which was maintained for up to 15 months. Users with higher baseline HbA1c showed greater reductions. Seventy‐five percent of users tracked their blood glucose values consistently during the whole observation period.

**Conclusion:**

These real‐world evidence findings demonstrate the effectiveness of a mobile diabetes app in improving glycemic control over an extended period. While statistical adjustments addressed potential biases, missing data remain a challenge. Further research, including controlled studies, is needed to confirm these real‐world results and further explore the underlying mechanisms of sustained HbA1c improvement.

## 1. Introduction

For insulin‐treated people with diabetes, digital diabetes management tools have become a relevant therapy component to improve patients’ self‐management and ultimately diabetes‐related health outcomes [1]. Glycemic control has been shown to prevent both hypo‐ and hyperglycemic events, thereby lowering the risk of diabetes‐related micro‐ and macrovascular complications, like diabetic retinopathy or neuropathy and cardiovascular diseases [2, 3].

Studies have demonstrated that blood glucose monitoring (BGM) in patients with both type 1 (T1D) and type 2 diabetes (T2D) mellitus is associated with improved glycemic control. Continuous blood glucose monitors (CGMs) have been shown to increase the time in range and decrease HbA1c levels [4]. However, (structured) BGM remains a time‐ and cost‐efficient alternative that can be as effective for managing diabetes as CGMs [7–9]; for example, the self‐monitoring of blood glucose (SMBG) has been shown to improve the understanding of blood glucose trends and enhance the precision of medical therapy decisions and adjustments [8].

This retrospective, observational study is aimed at analyzing real‐world data (RWD) from a digital device (ESYSTA, Emperra GmbH E‐Health Technologies, Germany) that incorporates both CGM and BGM data to enhance the glycemic control and self‐management of insulin‐treated people with diabetes. Its efficacy has been shown in a previous RCT and a controlled real‐world study [10, 11]. While RCTs remain the gold standard to evaluate the efficacy of medical devices and generate evidence, RWD (i.e., patients’ health data that are routinely collected independent of clinical trials) is becoming increasingly important in regulatory decisions [11], especially because it represents the daily management in real‐world care conditions that differ from controlled RCT settings.

The current, complementary real‐world approach is aimed at evaluating the effectiveness of ESYSTA based on a reduction of HbA1c levels.

## 2. Methods

### 2.1. Study Design

The present study is a retrospective, observational study, including users of ESYSTA. The study was preregistered in the Open Science Framework [12]. To report results in this manuscript, we followed the STROBE reporting guidelines for observational studies.

### 2.2. Intervention

ESYSTA is a digital health solution consisting of an app and a web portal. It was developed by Emperra GmbH E‐Health Technologies (Germany) to support adult insulin‐treated patients with diabetes to improve patient’s individual health status (reduction of HbA1c value) through enhanced diabetes self‐management. Generally, ESYSTA can be prescribed in Germany to insulin‐treated people with diabetes by their healthcare professionals.

By integrating data from both (continous) blood glucose meter (BGM) and insulin devices, as well as providing continuous feedback, ESYSTA bridges the gap between structured SMBG and continuous glucose monitoring. This comprehensive approach is aimed at empowering patients to optimize their glycemic control. This is in line with current guidelines that emphasize the importance of glycemic control to reduce the risk of diabetes‐related complications [13–17]. Relevant health data like glucose readings and insulin dosages can be transferred to the web‐based ESYSTA portal and the ESYSTA app either manually or automatically. Special algorithms continuously analyze and evaluate the data. A traffic light algorithm provides a quick overview of critical values or incorrect doses. The visualized and evaluated health data can be accessed via the ESYSTA portal and the ESYSTA app to help patients make informed decisions about their diabetes management. The individual patient may also grant the medical care team access to the ESYSTA portal to improve communication between the healthcare provider and patient, thus potentially allowing for remote treatment. Finally, the software supports patients with automatically generated support messages and suggestions for individual tailored prevention offers.

A detailed description of the functions of the ESYSTA app and ESYSTA portal is presented in Table S1.

### 2.3. Study Participants

Study participants are ESYSTA users who used the app until 23.11.2023 (date of data transfer). For the statistical analyses, it is necessary that changes in HbA1c can be calculated. As a result, only ESYSTA app users for whom at least two HbA1c values after initiation of ESYSTA app use are available within the 12‐month observation period, were included in the analysis. That is, a baseline value approximately within the time of starting to use ESYSTA and at least one more value in one of the following quarters had to be available.

Available HbA1c values are generally provided by the attending physicians via the ESYSTA portal. However, their provision is noncompulsory, and as such, HbA1c values are not available for every patient. HbA1c values are congruent with register data from the German diabetes management program, guaranteeing standardized HbA1c measurement procedures [18, 19].

### 2.4. Ethical Considerations

When using ESYSTA, all users give consent that their data may be used in an anonymized form for scientific analyses.

### 2.5. Statistical Analysis

The baseline HbA1c value was defined as being as close as possible to the start date of the initiation of ESYSTA app usage. Therefore, for the definition of the baseline HbA1c value, the following criteria were applied:

1. HbA1c value within a month (defined as 30 days) before or after initiation of ESYSTA app usage, if not available in the ESYSTA portal
2. HbA1c in the months (defined as ± 30 days) before initiation of ESYSTA app usage (defined as up to 120 days before), if not available in the ESYSTA portal
3. Closest value before/after initiation of ESYSTA app usage within 9 months after initiation of ESYSTA app usage (to still be able to at least calculate the change at 12 months)

The statistical model to be used for the HbA1c analysis is an analysis of variance with repeated measures. For this, a linear mixed model will be fitted with HbA1c change from baseline as the independent variable. The time (as the within‐subject factor corresponding to 3, 6, 9, 12, and 15 months) will be included as a fixed main effect. HbA1c (in percentage points) at baseline will be included to control for confounding, also in interaction with time. This interaction will be included and analyzed because the importance of the baseline will usually decrease over time. Additionally, to account for individual differences, a random intercept for each individual will be used. HbA1c changes after 6 and 12 months will be tested against an expected reduction of 0.4% with function *emmeans::test*. For testing, one‐sided *t*‐tests were used to test for clinically relevant HbA1c reductions, as is recommended in regulatory settings [20]. The minimal important difference of 0.4% was chosen slightly above the 0.3% threshold recommended by the European Medicines Agency (EMA) as clinically relevant to ensure a conservative definition of meaningful HbA1c improvement [21].

For the primary confirmatory analysis, missing data will be assumed to be missing at random (MAR). In the case of HbA1c values, assume that MAR might be reasonable since data points are noncompulsory and voluntarily provided by healthcare professionals. Hence, the availability of HbA1c is partly independent of the individual patient/user. As a sensitivity analysis, missing data will be imputed based on the last observation carried forward (LOCF) approach with the R package *zoo*, that is, missing values will be imputed based on the last available observed value. This process assumes that the course of the disease does not improve or worsen. In the case of the current study, this means that a patient, who no longer provides HbA1c values, will have the last available HbA1c value also for the next assessment time point(s).

All analyses were performed using the software R Version 4.3 and followed the preregistered analysis plan [12].

## 3. Results

**Figure 1:**
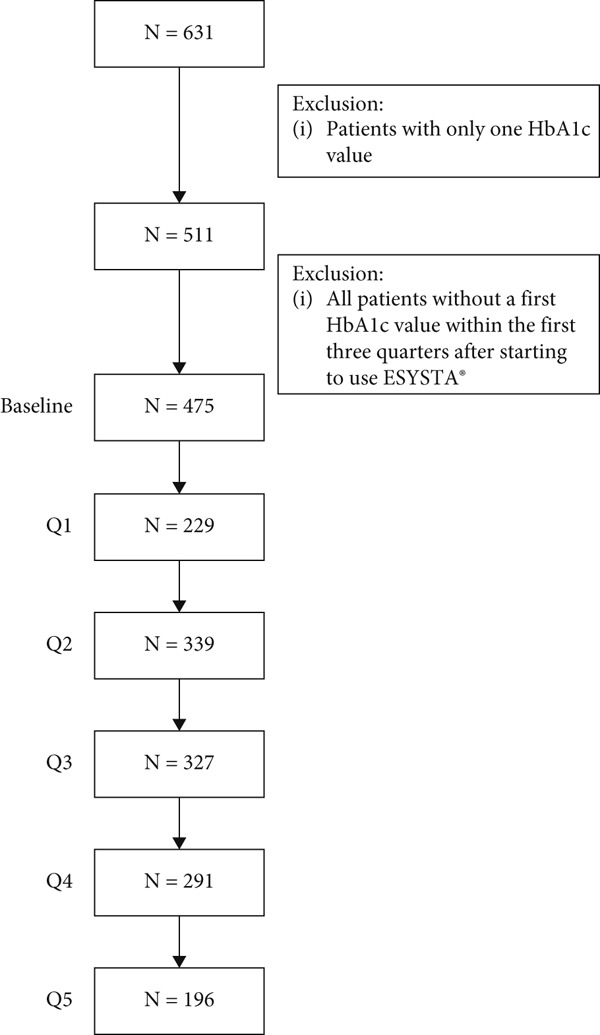
Patient flow chart.

At the time of analysis, data from 9.784 ESYSTA app users was available, including 631 with available HbA1c values in the ESYSTA portal. The first HbA1c value is from 04.03.2011 and the last from 23.11.2023. After defining the baseline value and the consecutive months, 478 ESYSTA app users had at least two HbA1c values in the time range of interest (i.e., up to one 15 months after initiation of ESYSTA app usage) and were included in the analyses. The exact patient flow and the number of ESYSTA app users over time (calculated based on the start date of using ESYSTA app) for which an HbA1c change could be calculated are shown in Figure 1.

### 3.1. Baseline Characteristics

The 475 ESYSTA app users, who are included in the analyses, had an average baseline HbA1c value of 8.26*%* ± 1.54*%* (5.0, 14.8) with approximately half of the patients having low baseline HbA1c levels (≤ 8%, *n* = 248). The average baseline HbA1c value of the group with low baseline HbA1c values (≤ 8%) was 7.13*%* ± 0.70*%* and for the group with high baseline HbA1c values (> 8%) was 9.45*%* ± 1.25*%*. Since ESYSTA is a German software, patients are from all over Germany.

For 313 patients, a baseline BMI was available, which was on average 32.13 ± 6.24 kg/m^2^, falling into the range of 18.52 to 51.31 kg/m^2^. The BMI at baseline between the high and low baseline HbA1c groups did not differ (32.09 ± 6.29 kg/m^2^ [≤ 8%] vs. 32.16 ± 6.22 kg/m^2^ [> 8%]).

Information on the indication was only available for 240 patients. As a result, the type of diabetes could be confirmed only for 124 T1D patients and 116 T2D patients. Since the indication was not added as a covariate, the availability did not affect whether or not people were included in the analysis.

No difference in baseline or BMI was observed between the patients with available information on indication, or even the different indications (T1D/T2D), while the HbA1c baseline levels of T2D patients were slightly higher (Table 1).

**Table 1:** Baseline HbA1c (in percentage) and BMI (kilograms per square meter) by indication.

**Indication**	**T1D (** **n** = 124**)**	**T2D (** **n** = 116**)**	**Information not available (** **n** = 338**)**
Baseline BMI	34.09 (6.7)	32.40 (5.3)	31.7 (6.6)
Baseline HbA1c	8.23 (1.1)	8.70 (1.4)	8.12 (1.6)

### 3.2. Engagement With the App

**Figure 2:**
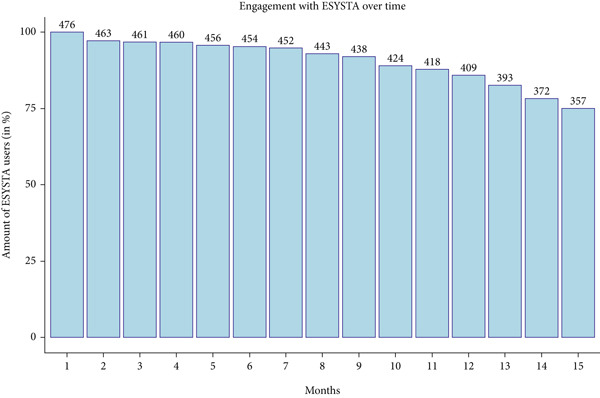
Number of patients that track blood glucose values in ESYSTA over time.

The available HbA1c levels for the ESYSTA users included in the study decreasing over time, since it is not mandatory for healthcare professionals to enter HbA1c levels of patients into the ESYSTA portal. The engagement with the ESYSTA app by the users is independent from it and, in fact, shows that most patients still use the app to track blood glucose after 1 year (86% in Month 12 and 75% in Month 15; see Figure 2). Overall, patients use the ESYSTA app to track blood glucose values almost daily (i.e., on average 3.11 ± 1.5 blood glucose trackings per day).

### 3.3. Change in HbA1c Values

On average, the HbA1c value of ESYSTA app users decreased by 0.6 percentage points after 6 months. The long‐term data show that this reduction was maintained (at 12 months) and further decreased (at 15 months).

**Figure 3:**
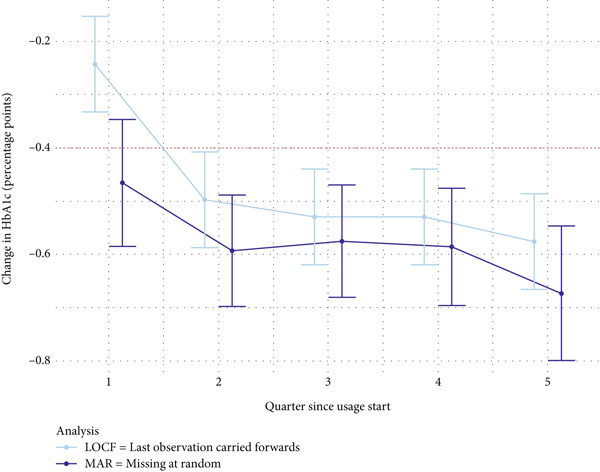
Estimated marginal means and 95% confidence intervals for the HbA1c reduction based on the LOCF and MAR analyses.

In the linear model fitted to assess the changes in HbA1c, only the interaction between time and baseline HbA1c (*X* ^2^(4) = 16.5339, *p* = 0.002) as well as the baseline HbA1c value (*X* ^2^(1) = 372.0334, *p* < 0.001) is significant. This indicates greater reductions with higher baseline HbA1c values over time. The average reduction of HbA1c values based on the linear mixed model is presented in Table 2 and Figure 3.

**Table 2:** Estimated marginal means and 95% confidence intervals for the HbA1c reduction over time according to MAR or and LOCF analyses.

**Months**	**HbA1c change**	**df**	**t** **-ratio**	**p** **value**	**d**
Missing at random (MAR)					
3	−0.47 [−0.59; −0.35]				
6	−0.59 [−0.70; −0.49]	1020	−3.629	< 0.001 ^∗∗∗^	−0.23 [−0.35, −0.10]
9	−0.56 [−0.68; −0.47]				
12	−0.59 [−0.70; −0.48]	1116	−3.314	< 0.001 ^∗∗∗^	−0.20 [−0.32, −0.08]
15	−0.67 [−0.80; −0.55]				

Last observation carried forward (LOCF)					
3	−0.24 [−0.33; −0.15]				
6	−0.50 [−0.59; −0.41]	1004	−2.132	0.017 ^∗^	−0.13 [−0.26, −0.01]
9	−0.53 [−0.62; −0.44]				
12	−0.53 [−0.62; −0.44]	1004	−2.851	0.002 ^∗∗^	−0.18 [−0.30, −0.06]
15	−0.58 [−0.67; −0.49]				

*Note:* Significance codes:  ^∗∗∗^< 0.001;  ^∗∗^< 0.01;  ^∗^< 0.05; < 0.1.

When testing whether a clinically relevant reduction in HbA1c of at least 0.4 percentage points was achieved after 6 and 12 months, one‐sided *t*‐tests were used. The results, summarized in Table 2, show that this goal was achieved at both time points, regardless of the analysis method used (MAR and LOCF).

### 3.4. Subgroup Analyses

Following the results of the linear mixed models, it can be assumed that the baseline HbA1c value has a strong influence on the trajectory of HbA1c levels while using ESYSTA. Hence, a subgroup analysis between patients with low and high baseline HbA1c levels was performed under the MAR assumption. For this, a second model was fitted that included the baseline HbA1c level as a categorical instead of a continuous variable. To categorize patients, glycemic treatment targets were used for orientation. While HbA1c levels of 7% (53 mmol/mol) or lower are generally considered a good target, individual patients might have HbA1c targets below 6.5% (48 mmol/mol) or even between 7.5% and 8% (58–64 mmol/mol) [22]. To include all possible acceptable targets, we defined the group with low baseline HbA1c levels within these targets (≤ 8%) and a group with high baseline HbA1c levels above these targets (> 8%).

In the MAR model, the main factor, time (*X* ^2^(4) = 8.396, *p* = 0.078), again is marginally significant, and the interaction between time and baseline HbA1c class (*X* ^2^(4) = 19.849, *p* = 0.001) as well as the baseline HbA1c class (*X* ^2^(1) = 104.195, *p* < 0.001) are significant. This indicates greater reductions in the subgroup with higher baseline HbA1c values (> 8%) compared to the subgroup with lower baseline HbA1c values (≤ 8%) over time. But there is no difference in HbA1c reductions over time within each baseline HbA1c group, that is, the reduction remains consistent within each group.

In the LOCF model, the main factor, time (*X* ^2^(4) = 81.383, *p* < 0.001), the interaction between time and baseline HbA1c (*X* ^2^(4) = 194.742, *p* < 0.001), and the baseline HbA1c class (*X* ^2^(1) = 358.998, *p* < 0.001) are significant. Again, this indicates higher reductions over time in the group with higher baseline HbA1c values (> 8%).

The average reduction in HbA1c values for both models based on the linear mixed model is presented in Table 3.

**Table 3:** Estimated marginal means and 95% confidence intervals for the HbA1c reduction by HbA1c baseline class over time and test statistics (based on the missing at random analysis).

**Months**	**HbA1c change**	**df**	**t** **-ratio**	**p** **value**	**d**
≤ 8%					
3	−0.04 [−0.23; 0.15]				
6	−0.10 [−0.27; 0.07]	958	3.480	1.000	0.22 [0.10, 0.35]
9	−0.06 [−0.22; 0.11]				
12	0.02 [−0.15; 0.20]	1029	4.659	1.000	0.29 [0.17, 0.42]
15	0.06 [−0.14; 0.25]				

> 8%					
3	−0.83 [−1.01; −0.64]				
6	−1.03 [−1.2; −0.87]	822	−7.536	< 0.001 ^∗∗∗^	−0.53 [−0.66, −0.39]
9	−1.03 [−1.2; −0.86]				
12	−1.14 [−1.31; −0.97]	931	−8.403	< 0.001 ^∗∗∗^	−0.55 [−0.68, −0.42]
15	−1.31 [−1.51; −1.12]				

*Note:* Significance codes:  ^∗∗∗^< 0.001;  ^∗∗^< 0.01;  ^∗^< 0.05; < 0.1.

When testing whether or not a clinically relevant reduction in HbA1c of at least 0.4 percentage points was achieved after 6 and 12 months, one‐sided *t*‐tests were used. The results show that the subgroup with high baseline HbA1c values achieved a clinically significant HbA1c reduction of at least 0.4 percentage points. In fact, the average reduction was approx. 1.0 percentage points at 6 and 12 months (see Table 3). The subgroup with lower HbA1c levels did not show a clinically significant reduction, showing stable HbA1c values over time with a maximal average reduction of 0.1 percentage points at 6 months and unchanged HbA1c values after 12 months (see Table 3).

Pairwise contrasts were used to quantify the difference between baseline HbA1c groups (see Table 4). The results showed that the subgroups significantly differed and the group with higher baseline HbA1c levels reduced their HbA1c levels on average by 1 percentage point more.

**Table 4:** Estimated marginal mean differences between baseline HbA1c groups at 6 and 12 months and test statistics (based on the MAR analysis).

**Months**	**Difference in HbA1c change**	**df**	**t** **-ratio**	**p** **value**	**d**
6	−0.93 [−1.17; −0.7]	891	−7.736	< 0.001 ^∗∗∗^	−0.52 [−0.65, −0.38]
12	−1.16 [−1.41; −0.91]	981	−9.233	< 0.001 ^∗∗∗^	−0.59 [−0.72, −0.46]

*Note:* Significance codes:  ^∗∗∗^< 0.001;  ^∗∗^< 0.01;  ^∗^< 0.05; < 0.1.

## 4. Discussion

The aim of our analyses was to evaluate whether the use of ESYSTA leads to improvements in self‐management and in glycemic control in insulin‐treated people with diabetes, expressed by a change in HbA1c (in percentage points). Overall, a continuous reduction in HbA1c levels was observed, supporting the hypothesis that a clinically relevant reduction of at least 0.4 percentage points can be achieved after 6 months and maintained. In fact, patients on average reduced their HbA1c levels by −0.6 percentage points. These results are further reinforced by a more conservative sensitivity analysis, which imputed missing data using the LOCF method. Further, the number of blood glucose tracking of patients showed a stable and daily engagement with ESYSTA over time.

When compared with other studies that evaluated the treatment effect of ESYSTA, the changes in HbA1c after 6 months are comparable to those in a RCT (−0.48 [−0.66; −0.29]) [9] and nearly identical to another real‐world study with a matched control group that showed changes in the intervention group (ESYSTA users) after 6 and 12 months of −0.60 percentage points (−0.79; −0.42) and −0.66 percentage points (−0.85; −0.46) [10].

The reduction in HbA1c observed in ESYSTA users is comparable to or even better than that reported in other one‐arm real‐world studies of similar digital interventions, for example, the *integrated personalized diabetes management* program. It was analyzed in a one‐arm study with insulin‐treated T1D and T2D patients and showed similar average reductions after 6 months of 0.5*%* ± 0.6 percentage points (*p* = 0.045, *d* = 0.81) [23]. A retrospective analysis of *mySugr* users showed average HbA1c reductions of −0.4 percentage points after approximately 3 months [24].

### 4.1. Strengths and Limitations

Overall, the current analysis demonstrates strong external validity, as it is based on data from real‐world users in an actual care setting. In most trials using RWD, the selection process of the data, the handling of missing data, and the choice of appropriate statistical methods to address these issues are key factors contributing to low evidence quality [25]. To increase the quality of evidence, the planned statistical analysis was preregistered including details of the data selection process and the handling of missing data [12]. Moreover, the outcome data used is not self‐reported but instead provided by healthcare professionals, which helps minimize the potential for reporting bias related to HbA1c.

Two different approaches to handling missing data were applied to enhance the validity of the results. Since the more conservative imputation method (LOCF) yielded similar outcomes compared to the less conservative MAR approach, the assumption of MAR can be considered valid. This is further supported by the engagement data of users, which demonstrated stable usage over time. While fewer HbA1c values are available for patients over time, most patients still track blood glucose values in the ESYSTA app after 12 and 15 months (around 80%). This reinforces the validity of the results, as consistent app usage indicates that the missing data does not stem from users discontinuing the app.

Additional missing patient information (e.g., gender, age, and indication) remains a main source of bias. However, an additional analysis including only patients with available indication data showed no differences based on the type of diabetes. Instead, the linear mixed models, suggested an assosciation the effect of changes in HbA1c and baseline HbA1c levels (see Table S2). Subgroup analyses were consistend with the findings that patients with higher baseline HbA1c levels experienced significantly greater reductions in HbA1c over time, with average decreases of around 1 percentage point. This trend of greater HbA1c reductions in patients with higher baseline levels has been observed in several previous real‐world studies [23, 25]. Especially extreme or high measurements are likely to be lower when measured again over time due to natural variation. These statistical phenomena, known as regression to the mean (RTM), can lead to incorrect conclusions about the effectiveness of an intervention by mistakenly attributing natural fluctuation in the population (i.e., RTM) to intervention effects. To rule out that the observed effect is merely due to natural fluctuations, an RTM detection method was applied [26, 27]. The results indicate that the observed changes in HbA1c levels represent a true intervention effect, rather than an RTM (see Figure S1).

## 5. Conclusions

Overall, the present real‐world study supports previous evidence from an RCT and a real‐world study with a matched controlled group that the digital device ESYSTA is associated with significant and continuous reductions in HbA1c levels in T1D and T2D insulin‐treated people with diabetes. We tried to mitigate potential biases inherent in RWD by employing appropriate statistical methods. However, missing values remain a persistent challenge in RWD analyses.

## Conflicts of Interest

P.E.H.S. works as a scientific consultant and principal investigator for the Emperra GmbH E‐Health Technologies and the present study. L.R. and J.S. declare that the research was conducted in the absence of any commercial or financial relationships that could be construed as a potential conflict of interest.

## Author Contributions

Lena Roth was responsible for data curation, formal analysis, investigation, methodology, validation, visualization, and the writing and reviewing of the draft. Julian Stein supported the conduction and visualization of results of the explorative analysis and the review of the manuscript. Peter E. H. Schwarz was responsible for the conceptualization, funding acquisition, project administration, supervision, validation, and review of the manuscript.

## Funding

The author(s) declare that financial support was received for the research and/or publication of this article. The study was funded by the Vision2b GmbH.

## Supporting information


**Supporting Information** Additional supporting information can be found online in the Supporting Information section. Table S1: Description of functions of ESYSTA app and portal. Table S2: Results of the explorative analysis model including the covariance indication (type of diabetes). Figure S1: Results of the detection of potential regression to the mean.

## Data Availability

The original contributions presented in the study are included in the article and its Supporting Information. Further inquiries can be directed to the corresponding author.

## References

[1] ElSayed N. A. , Aleppo G. , Aroda V. R. , Bannuru R. R. , Brown F. M. , Bruemmer D. , Collins B. S. , Hilliard M. E. , Isaacs D. , Johnson E. L. , Kahan S. , Khunti K. , Leon J. , Lyons S. K. , Perry M. L. , Prahalad P. , Pratley R. E. , Seley J. J. , Stanton R. C. , Gabbay R. A. , and on behalf of the American Diabetes Association , 7. Diabetes Technology: Standards of Care in Diabetes—2023, Diabetes Care. (2023) 46, no. Supplement_1, S111–S127, 10.2337/dc23-S007, 36507635. 36507635 PMC9810474

[2] Lachin J. M. , Genuth S. , Nathan D. M. , Zinman B. , Rutledge B. N. , and DR Group , Effect of Glycemic Exposure on the Risk of Microvascular Complications in the Diabetes Control and Complications Trial—Revisited, Diabetes. (2008) 57, no. 4, 995–1001, 10.2337/db07-1618, 2-s2.0-42449121829. 18223010

[3] Stratton I. M. , Association of Glycaemia With Macrovascular and Microvascular Complications of Type 2 Diabetes (UKPDS 35): Prospective Observational Study, BMJ. (2000) 321, no. 7258, 405–412, 10.1136/bmj.321.7258.405. 10938048 PMC27454

[4] Lin R. , Brown F. , James S. , Jones J. , and Ekinci E. , Continuous Glucose Monitoring: A Review of the Evidence in Type 1 and 2 Diabetes Mellitus, Diabetic Medicine. (2021) 38, no. 5, e14528, 10.1111/dme.14528. 33496979

[5] Janapala R. N. , Jayaraj J. S. , Fathima N. , Kashif T. , Usman N. , Dasari A. , Jahan N. , and Sachmechi I. , Continuous Glucose Monitoring Versus Self-Monitoring of Blood Glucose in Type 2 Diabetes Mellitus: A Systematic Review With Meta-Analysis, Cureus. (2019) 11, no. 9, e5634, 10.7759/cureus.5634, 31700737. 31700737 PMC6822918

[6] Jancev M. , Vissers T. A. C. M. , Visseren F. L. J. , van Bon A. C. , Serné E. H. , DeVries J. H. , de Valk H. W. , and van Sloten T. T. , Continuous Glucose Monitoring in Adults With Type 2 Diabetes: A Systematic Review and Meta-Analysis, Diabetologia. (2024) 67, no. 5, 798–810, 10.1007/s00125-024-06107-6, 38363342. 38363342 PMC10954850

[7] Bergenstal R. M. , Mullen D. M. , Strock E. , Johnson M. L. , and Xi M. X. , Randomized Comparison of Self-Monitored Blood Glucose (BGM) Versus Continuous Glucose Monitoring (CGM) Data to Optimize Glucose Control in Type 2 Diabetes, Journal of Diabetes and its Complications. (2022) 36, no. 3, 108106, 10.1016/j.jdiacomp.2021.108106, 35131155. 35131155

[8] Chircop J. , Sheffield D. , and Kotera Y. , Systematic Review of Self-Monitoring of Blood Glucose in Patients With Type 2 Diabetes, Nursing Research. (2021) 70, no. 6, 487–497, 10.1097/NNR.0000000000000542. 34292228

[9] Roth L. , Steckhan N. , and Schwarz P. E. H. , Impact of a Digital Application on HbA1c Levels in Insulin-Treated Diabetes Patients: A Randomized Controlled Study, in submission. 10.3389/fdgth.2025.1544668 PMC12257309 40661654

[10] Roth L. , Wagner C. J. , Petra R. , Birgit K. , Steckhan N. , and Schwarz P. E. H. , Evaluation of a Digital Health Application for Diabetics Under Real World Conditions: Superior Outcomes Compared to Usual Care in a Matched Controlled Study, Diabetology. (2025) 6, no. 9, 10.3390/diabetology6090085.

[11] Cave A. , Kurz X. , and Arlett P. , Real-World Data for Regulatory Decision Making: Challenges and Possible Solutions for Europe, Clinical Pharmacology & Therapeutics. (2019) 106, no. 1, 36–39, 10.1002/cpt.1426, 2-s2.0-85064152031. 30970161 PMC6617710

[12] Roth L. , Stein J. , and Schwarz P. E. H. , Real-World Evidence of a Digital Application for Insulin-Treated Diabetes Patients, 2025, in submission.

[13] Bundesärztekammer (BÄK) , Kassenärztliche Bundesvereinigung (BKV) , and Arbeitsgemeinschaft der Wissenschaftlichen Medizinischen Fachgesellschaften (AWMF) , Nationale Versorungsleitlinie Typ-2-Diabetes - Teilpublikation der Langfassung, 2. Auflage. Version 1, 2021, 10.6101/AZQ/000475.

[14] Buse J. B. , Wexler D. J. , Tsapas A. , Rossing P. , Mingrone G. , Mathieu C. , D’Alessio D. A. , and Davies M. J. , 2019 Update to: Management of Hyperglycemia in Type 2 Diabetes, 2018. A Consensus Report by the American Diabetes Association (ADA) and the European Association for the Study of Diabetes (EASD), Diabetes Care. (2020) 43, no. 2, 487–493, 10.2337/dci19-0066, 31857443. 31857443 PMC6971782

[15] Cosentino F. , Grant P. J. , Aboyans V. , Bailey C. J. , Ceriello A. , Delgado V. , Federici M. , Filippatos G. , Grobbee D. E. , Hansen T. B. , Huikuri H. V. , Johansson I. , Jüni P. , Lettino M. , Marx N. , Mellbin L. G. , Östgren C. J. , Rocca B. , Roffi M. , Sattar N. , Seferović P. M. , Sousa-Uva M. , Valensi P. , Wheeler D. C. , and ESC Scientific Document Group , 2019 ESC Guidelines on Diabetes, Pre-Diabetes, and Cardiovascular Diseases Developed in Collaboration With the EASD, European Heart Journal. (2020) 41, no. 2, 255–323, 10.1093/eurheartj/ehz486, 31497854. 31497854

[16] Davies M. J. , Aroda V. R. , Collins B. S. , Gabbay R. A. , Green J. , Maruthur N. M. , Rosas S. E. , del Prato S. , Mathieu C. , Mingrone G. , Rossing P. , Tankova T. , Tsapas A. , and Buse J. B. , Management of Hyperglycemia in Type 2 Diabetes, 2022. A Consensus Report by the American Diabetes Association (ADA) and the European Association for the Study of Diabetes (EASD), Diabetes Care. (2022) 45, no. 11, 2753–2786, 10.2337/dci22-0034, 36148880. 36148880 PMC10008140

[17] Deutsche Diabetes Gesellschaft (DDG) , S3-Leitlinie Therapie des Typ-1-Diabetes. Version 2.0. AWMF-Register-Nr. 057-024, 2023, Accessed: Jun. 06, 2024. [Online]. Available: https://register.awmf.org/assets/guidelines/057-013l_S3-Therapie-Typ-1-Diabetes_2018-08.pdf.

[18] Institut für angewandte Sozialwissenschaft GmbH (INFAS) , Bericht der strukturierten Behandlungsprogramme der gesetzlichen Krankenkassen – Indikation Diabetes mellitus Typ 2 Erstellt durch infas und MNC, Die Gesetzlichen Krankenkassen, 2024. Accessed: Jun. 26, 2024. [Online]. Available: https://www.barmer.de/resource/blob/1023672/de3e2ccb334e697436e2e226c2e5f144/besser-leben-programm-evaluationsbericht-infas-diabetes-typ2-data.pdf.

[19] Zentralinstitut für die kassenärztliche Versorgung in der Bundesrepublik Deutschland , DMP-Atlas Nordrhein-Westfalen. Regionalisierte Darstellung der Disease-Management-Programme, 2024, Accessed: Oct. 07, 2024. [Online]. Available: https://www.zi-dmp.de/dmp-atlas_nrw/dmp_d2_adt.html.

[20] European Medicines Agency (EMA) , ICH Topic E 9: Statistical Principles for Clinical Trials, 1998, Accessed: Feb. 09, 2024. [Online]. Available: https://www.ema.europa.eu/en/documents/scientific-guideline/ich-e-9-statistical-principles-clinical-trials-step-5_en.pdf.

[21] European Medicines Agency (EMA) , Guideline on Clinical Investigation of Medicinal Products in the Treatment or Prevention of Diabetes Mellitus, 2012. 10.1007/s00125-024-06162-z 38702529

[22] ElSayed N. A. , Aleppo G. , Aroda V. R. , Bannuru R. R. , Brown F. M. , Bruemmer D. , Collins B. S. , Hilliard M. E. , Isaacs D. , Johnson E. L. , Kahan S. , Khunti K. , Leon J. , Lyons S. K. , Perry M. L. , Prahalad P. , Pratley R. E. , Seley J. J. , Stanton R. C. , Gabbay R. A. , and on behalf of the American Diabetes Association , 6. Glycemic Targets: Standards of Care in Diabetes—2023, Diabetes Care. (2023) 46, no. Supplement_1, S97–S110, 10.2337/dc23-S006, 36507646. 36507646 PMC9810469

[23] Mora P. , Buskirk A. , Lyden M. , Parkin C. G. , Borsa L. , and Petersen B. , Use of a Novel, Remotely Connected Diabetes Management System Is Associated With Increased Treatment Satisfaction, Reduced Diabetes Distress, and Improved Glycemic Control in Individuals With Insulin-Treated Diabetes: First Results From the Personal Diabetes Management Study, Diabetes Technology & Therapeutics. (2017) 19, no. 12, 715–722, 10.1089/dia.2017.0206, 2-s2.0-85038618260, 29027812. 29027812 PMC5734194

[24] Fredrick D. , Harald M. , and Johanna K. , Real-World Assessments of mySugr Mobile Health App, Diabetes Technology & Therapeutics. (2019) 21, no. S2, S2-35–S2-40, 10.1089/dia.2019.0019, 2-s2.0-85066995303, 31169427. 31169427

[25] Bodner E. , Roth L. , Wiencke K. , Bischoff C. , and Schwarz P. E. , Effect of Multimodal App-Based Interventions on Glycemic Control in Patients With Type 2 Diabetes: Systematic Review and Meta-Analysis, Journal of Medical Internet Research. (2025) 27, no. 1, e54324, 10.2196/54324. 39854703 PMC11806272

[26] Timpel P. , Oswald S. , Schwarz P. E. H. , and Harst L. , Mapping the Evidence on the Effectiveness of Telemedicine Interventions in Diabetes, Dyslipidemia, and Hypertension: An Umbrella Review of Systematic Reviews and Meta-Analyses, Journal of Medical Internet Research. (2020) 22, no. 3, e16791, 10.2196/16791, 32186516. 32186516 PMC7113804

[27] Linden A. , Assessing Regression to the Mean Effects in Health Care Initiatives, BMC Medical Research Methodology. (2013) 13, no. 1, 10.1186/1471-2288-13-119, 2-s2.0-84884656212, 24073634. PMC3849564 24073634

[28] Ostermann T. , Willich S. N. , and Lüdtke R. , Regression Toward the Mean – A Detection Method for Unknown Population Mean Based on Mee and Chua’s Algorithm, BMC Medical Research Methodology. (2008) 8, no. 1, 10.1186/1471-2288-8-52, 2-s2.0-50649122718, 18687143. PMC2527023 18687143

