# Supplementary material for: Real‐World Evidence on Mobile App–Supported Diabetes Management in Insulin‐Treated Patients
Source: J Diabetes Res. 2025 Dec 29;2025:6671361. doi: 10.1155/jdr/6671361 (PMC12767227; doi:10.1155/jdr/6671361)
Supplement: Supplementary file 1 — Supporting Information Additional supporting information can be found online in the Supporting Information section. Table S1: Description of functions of ESYSTA app and portal. Table S2: Results of the explorative analysis model including the covariance indication (type of diabetes). Figure S1: Results of the detection of potential regression to the mean. [file JDR-2025-6671361-s001.docx]

Supplement

Table of Contents

[Supplementary Table 1: Description of Functions of ESYSTA® APP and portal. 1](#_Toc190871353)

[Supplementary Table 2: Results of the explorative analysis model including the covariance indication 2](#_Toc190871354)

[Supplementary Figure 2: Detection of potential Regression to the Mean 3](#_Toc190871355)

# Supplementary Table 1: Description of Functions of ESYSTA® APP and portal.

| **ESYSTA® APP** | **ESYSTA® PORTAL** |
| --- | --- |
| - automatic data synchronization with the ESYSTA® PORTAL - fully automatic synchronization of limit values, insulin type used and measurement units (mmol/l and mg/dl) - display of diary data when no suitable computer is available to access the ESYSTA® PORTAL, e. g. on vacation or during doctor consultations - detailed daily display of blood glucose, insulin and bread units in a clearly arranged timeline diary - blood glucose history in three- and seven-day view - integrated diagram with insulin doses and blood glucose values - clear data evaluation/target range analysis - simplified display and preparation of selected therapy data for optimization and support of patient self-management - quick overview of the metabolic setting by the ESYSTA® Assistant with the ESYSTA® traffic light - manual recording of blood glucose readings, insulin doses and bread units - marking of blood glucose readings for assignment to meals (fasting, pre- and postprandial and other selections) - import of blood glucose and insulin data simply via Bluetooth®. - also, usable when offline | - worldwide usable, easy web access to therapy data via web browser - data preparation in clear tables and graphics – complete and without gaps - detailed daily display of blood glucose, insulin and bread units in a clearly arranged diary - daily blood glucose history in seven-day view - integrated diagram display of insulin doses and blood glucose values - clear data evaluation/target range analysis - marking of blood glucose readings to set them into context with meals (fasting, pre- and postprandial and further selection options) - daytime analysis of blood glucose values - simplified display of selected therapy data for optimization and support of patient self-management - data access for the physician possible - quick overview of the metabolic setting by the ESYSTA® APP with specially developed traffic light function supporting the patient in his/her therapy (empowerment) and gives the physician indications of a possible need for intervention - protected message exchange with the physician - display of relevant parameters for each patient and medical professional - entry of further medical or laboratory data in the form of an electronic patient file is possible - support for automated import of data from suitable blood glucose meters and insulin pens |

# Supplementary Table 2: Results of the explorative analysis model including the covariance indication

| Covariate | X^2^-statistic | Degrees of Freedom | p-value |
| --- | --- | --- | --- |
| Indication | 0.2334 | 1 | 0.62901 |
| Baseline HbA1c | 57.7687 | 1 | < 0.001*** |
| time | 5.6805 | 4 | 0.22431 |
| Time*indication | 2.5647 | 4 | 0.63309 |
| Time*Baseline HbA1c | 10.5420 | 4 | 0.03222* |

*Note: Signif. codes: 0 ‘***’ 0.001 ‘**’ 0.01 ‘*’ 0.05 ‘.’ 0.1 ‘ ’ 1*

# Supplementary Figure 1: Detection of potential Regression to the Mean


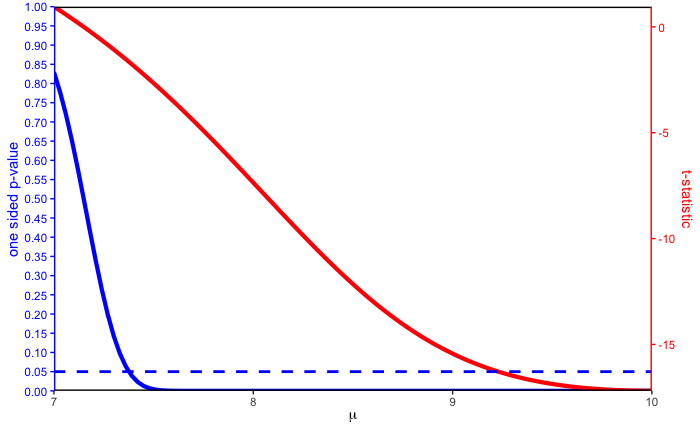
*Note: Under the assumption that the actual population HbA1c mean (μ) lays between 7% and 10%, a significant intervention effect (p ≤ 0.05) at 6 months can be assumed if the actual population mean (μ) is above equal or above 7.39%.*
